# A pH-responsive liposomal nanoplatform for co-delivery of a Pt(IV) prodrug and cinnamaldehyde for effective tumor therapy

**DOI:** 10.3389/fbioe.2023.1191534

**Published:** 2023-05-05

**Authors:** Ting Tang, Yufang Gong, Yuan Gao, Xinlong Pang, Shuangqing Liu, Yulan Xia, Dongsheng Liu, Lin Zhu, Qing Fan, Xiao Sun

**Affiliations:** ^1^ Department of Dental Implantology, Hefei Stomatology Hospital, Clinical School of Anhui Medical University, Hefei, China; ^2^ Department of Pharmacy, Shandong Cancer Hospital and Institute, Shandong First Medical University and Shandong Academy of Medical Sciences, Jinan, China

**Keywords:** Pt(IV) prodrug, cinnamaldehyde, liposome, reactive oxygen species, tumor synergistic therapy

## Abstract

**Introduction:** The tumor microenvironment (TME) is mainly characterized by abnormally elevated intracellular redox levels and excessive oxidative stress. However, the balance of the TME is also very fragile and susceptible to be disturbed by external factors. Therefore, several researchers are now focusing on intervening in redox processes as a therapeutic strategy to treat tumors. Here, we have developed a liposomal drug delivery platform that can load a Pt(IV) prodrug (DSCP) and cinnamaldehyde (CA) into a pH-responsive liposome to enrich more drugs in the tumor region for better therapeutic efficacy through enhanced permeability and retention effect.

**Methods:** Using the glutathione-depleting properties of DSCP together with the ROS-generating properties of cisplatin and CA, we synergistically altered ROS levels in the tumor microenvironment to damage tumor cells and achieve anti-tumor effects *in vitro*.

**Results:** A liposome loaded with DSCP and CA was successfully established, and this liposome effectively increased the level of ROS in the tumor microenvironment and achieved effective killing of tumor cells *in vitro*.

**Conclusion:** In this study, novel liposomal nanodrugs loaded with DSCP and CA provided a synergistic strategy between conventional chemotherapy and disruption of TME redox homeostasis, leading to a significant increase in antitumor effects *in vitro*.

## 1 Introduction

Tumor poses a severe threat to human health for its high rate of morbidity and mortality. Conventional antitumor drugs lack selectivity in killing tumor cells and frequently cause side effects on normal tissues and organs. Liposomes are miniature vesicle structures mainly composed of cholesterol and lecithin, and they have been widely studied as delivery systems for their potential in carrying drugs (Lee et al., 2015; Shah et al., 2020; Large et al., 2021; Park et al., 2022). In the aqueous phase space, liposomes can pack hydrophilic drugs in, and they can also be loaded with lipophilic drugs amidst the phospholipid bilayer (Zununi Vahed et al., 2017), which makes them an ideal drug carrier for various types of polar molecules. The surface of liposomes can also be modified with peptides, transmembrane proteins, and other components to improve the targeting capability, biocompatibility, and stability of liposomal nanodrugs. Such modifications allow the liposomes to disassemble responsively in the tumor microenvironment and apply the drugs in a higher accuracy (Di et al., 2020). For example, pH-responsive liposomes prepared by doping liposomes with pH-sensitive components could release drugs in a pH-responsive manner following their entry into the acidic TME, while releasing a less amount of drug in normal tissues at pH 7.4 (Brewer et al., 2015): the TME-responsive liposomes can reduce drug loss during delivery and premature drug release, decreasing drug toxicity to normal tissues. Other commonly used acid-responsive modifications, such as acid-responsive membrane-penetrating peptides, involve the conversion on molecules’ polarity under acidic conditions, leading to liposome depolymerization, which is also a prevailing approach to achieving acid-responsive release of drugs (Zhou et al., 2022). In addition to surface modifications, the optimization of liposome delivery systems can control drug release rate, prolong the duration of drug action, and enhance drug targeting ability (Guimarães et al., 2021). These optimizations endow liposome delivery systems with great potential in the diagnosis and treatment of diseases such as tumors (Au et al., 2016).

Reactive oxygen species (ROS) are a series of single-electron reduction products of oxygen inside the body, including superoxide anions, hydrogen peroxide, hydroxyl radicals, lipid peroxides and protein peroxides (Cheung and Vousden, 2022). ROS maintain dynamic homeostasis through several redox reactions in biological systems and participate as signaling molecules in cellular regulation (Yang et al., 2019; Zhang et al., 2019). When intracellular ROS level increases over the threshold, antitumor effects will be exerted to cells to induce apoptosis, cell necrosis and ferroptosis (Prasad et al., 2017; Moloney and Cotter, 2018; Wang et al., 2021a). Thus it will be turned into a promising approach to the prevention and eradication on tumor if we can accurately elicit ROS level elevation in cancer cells only, and there have been already some mature therapies, i.e., chemodynamic therapy (CDT). In recent years, it has been found that many traditional chemotherapeutic drugs can kill tumor cells by inducing oxidative stress; for example, the conventional chemotherapeutic drug cisplatin kills tumor cells by inducing oxidative stress in tumor cells to increase mitochondrial damage. However, cisplatin-induced oxidative stress affects not only cancer cells but also normal cells. For example, an excessive ROS production in cochlear cells may cause hearing loss, which is one of the common side effects of cisplatin (Ghosh, 2019). In addition, cisplatin-induced cytotoxicity is often mitigated by glutathione (GSH): when treated with cisplatin, cancer cells are in a state of oxidative stress and ROS levels are elevated. In order to restore their redox homeostasis, cells reactively activate a reduction system involving GSH to avoid cell death and induce cellular resistance (An et al., 2019; Sun et al., 2021). Therefore, researchers prefer to use low doses of cisplatin that have both targeting and GSH-depleting properties to improve the efficacy of cisplatin when keeping the side effects at a much low rate at the same time.

**SCHEME 1:**
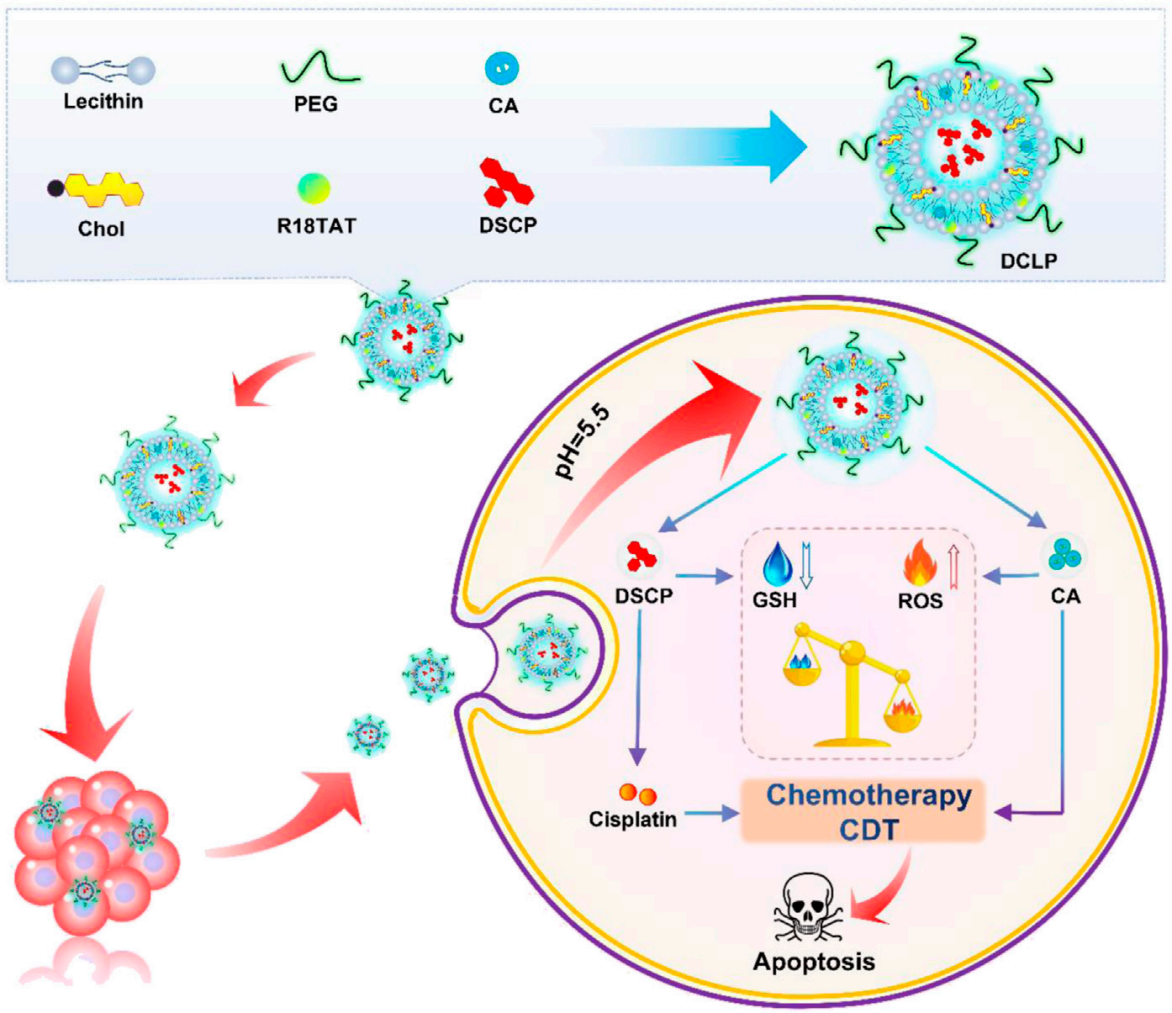
Schematic illustration of the nanotheranostic design of DCLP.

In the present study, we developed a TME-responsive, liposome-encapsulated nano-drug, DCLP, to achieve low-dose, highly effective chemotherapy. It also enhanced intracellular ROS at the same time to increase the oxidation level in the tumor microenvironment and inhibited the repair of cellular damage by the antioxidant system, causing a complete imbalance in the redox balance of the cells. This procedure could carry out a more ideal tumor cell killing effect. As shown in Scheme 1, to promote drug release, this nanodrug used liposomes as the main carrier and acid-reactive peptides added during the synthesis process, which can be reactively decomposed in the acidic TME. Two antitumor drugs, Disuccinatocisplatin (DSCP) and cinnamaldehyde (CA) were loaded Inside the liposomes. Previous studies have reported that CA is a food spice with antitumor effects and a reliable ROS generator: it can stimulate a high mitochondrial production of ROS to induce excessive oxidative stress in tumor cells, thereby activating the apoptotic pathway thereby activating the apoptotic pathway (Xu et al., 2018; Tang et al., 2019; Tu et al., 2022; Banerjee and Banerjee, 2023). However, it bears a short half-life, low bioavailability, and being oxidized quickly as a member of aldehyde group, all of these factors lead to a discontented outcome of the disease and tend to make its efficacy lower than that of other commonly used anticancer drugs, which restricts the clinical application of CA. DSCP is a tetravalent platinum compound obtained by modifying cisplatin. DSCP can only achieve pharmacological effects when it is converted into an active drug *in vivo*, which has the advantage of increasing drug stability and reducing toxic side effects. The reduction of DSCP to divalent cisplatin *in vivo* can function in multiple threads: firs it can deplete GSH, then promotes ROS accumulation at the same time, and further enhances the ROS-based cell killing effect. CA and DSCP can thus synergistically promote ROS accumulation and reduce GSH levels, enhance chemotherapeutic efficacy, and reduce tumor resistance to cisplatin (Shin et al., 2014). In summary, in the present study, a novel dual drug delivery nanosystem, named DCLP, was constructed. This nanosystem not only promoted ROS production through a simple and practical approach but also suppressed the antioxidant system in tumor cells to enhance ROS-mediated oxidative stress treatment with substantial antitumor effects.

## 2 Experimental section

### 2.1 Materials and preparation

Cisplatin (99%), succinic anhydride (99%), and CA (98%) were purchased from Shanghai Aladdin Bio-Chem Technology Co., Ltd. (Shanghai, China). Cholesterol (97%) and lecithin from soybean (60%) were purchased from Sangon Biotech Co., Ltd. (Shanghai, China). DSPE-PEG2000 (95%) was purchased from Shanghai Ponsure Biotech Co., Ltd. (Shanghai, China). C18-TAT peptide (RKK(C18)RRQRRR, 94.63%) was synthesized by China Peptides Co., Ltd. (Shanghai, China). The glutathione reductase/5,5ʹ-dithiobis-(2-nitrobenzoic acid) (DTNB) recycling assay kit was provided by Beyotime Biotechnology (Haimen, JiangSu, China). The MTT cell proliferation and cytotoxicity assay kit and the reactive oxygen species assay kit were obtained from Sangon Biotech Co., Ltd. Fetal bovine serum (FBS) was purchased from Gibco (United States). PBS and Dulbecco’s Modified Eagle’s Medium (DMEM) were purchased from Biological Industries Israel Beit-Haemek Ltd. (Kibbutz Beit-Haemek, Israel). Penicillin-streptomycin solution and recombinant trypsin-EDTA solution were provided by Beyotime Biotechnology. Other chemical reagents were purchased from Sinopharm Chemical Reagent Co., Ltd. (Shanghai, China). All the reagents were used as received without further purification. Through the whole procedure of the experiments, the water used was all deionized water with a resistivity higher than 18 MΩ cm.

### 2.2 Preparation of DSCP c,c,t-[Pt (NH_3_)_2_Cl_2_(O_2_CCH_2_CH_2_CO_2_H)_2_]

DSCP was synthesized by referring to previously published methods (Cheng et al., 2014; Wang et al., 2021b; Cheng et al., 2021): A total of 374 mg of cisplatin (1.25 mmol) was suspended in 15 mL of water, and 12.5 mL of 30% H_2_O_2_ (122.38 mmol) was added dropwise. This solution was then stirred at 55 °C for 1.5 h; it was subsequently cooled to room temperature and left to stand for 12 h. The solution was lyophilized, and the precipitate was washed three times each with cold water, acetone, and ether and dried again to yield the intermediate product oxoplatin (c,c,t-[PtCl_2_(OH)_2_(NH_3_)_2_]). Next, 300 mg succinic anhydride (3 mmol) was added to 3.5 mL N,N-dimethylformamide solution of oxoplatin (100 mg, 0.3 mmol), and the mixture was stirred at room temperature overnight. After lyophilization, the product was washed with chloroform and ether, lyophilized, and dried again to obtain a light-yellow solid. The product was then dried and used for subsequent experiments.

### 2.3 Isobolographic analysis

After determining the effective therapeutic doses of DSCP and CA, the synergistic effect of the two drugs, DSCP and CA, was tested in specifically selected 4T1 cells for the construction of the isobologram. The ratio of drug combinations was as 1:2, 2:1 and 1:1. After obtaining the combined drugs, the need for the combination of DSCP and CA was determined by calculating the amount of each of the two drugs compared to the drugs simply added together.

### 2.4 Synthesis of DCLP

DCLP liposomes were fabricated by the self-emulsifying solvent evaporation method as follows: soy lecithin, cholesterol, DSPE-PEG2000, C18TAT peptide, and CA were dissolved in 5 mL of chloroform in the molar ratio of 60:30:2:6:20. Chloroform was then removed by a rotary evaporator at 37 °C in vacuum to form a thin film. Trace amounts of chloroform were completely removed after subsequent drying in vacuum. The total amount of aqueous solution (including water in which the cisplatin precursor was dissolved/the amount of cisplatin added was 5 eq) was kept at 5 mL, and the vessel was placed in an ice bath at 120 W for 5 min (5 s continuous, 5 s interrupted) to obtain DCLP.

### 2.5 Characterization

^1^H NMR spectra of DSCP were measured using BRUKER AVANCE 400. The solvent used was (CD_3_)_2_SO. Chemical shifts were expressed in parts per million [ppm (δ)], and the hydrodynamic diameters and zeta potential were estimated using a Malvern Zetasizer Nano ZS. A 120 kV transmission electron microscope (TEM; Ruli TEM HT7800) was used to examine the morphology of DSCP liposomes. Cisplatin content in the liposomes was determined by ICP-OES (PerkinElmer 8300, Waltham, Massachusetts, United States). Fluorescence microscopy (EVOS™ M7000, Thermo Fisher Scientific) was used to observe the condition of drug-treated cells and stained cells.

### 2.6 Statistical analysis

All data are expressed as mean ± standard deviation (SD). Two-tailed Student’s t-test and one-way analysis of variance (ANOVA) with Tukey’s *post hoc* test were used to determine statistical significance between the groups. *p* < 0.05 was considered to be statistically significant.

## 3 Results

### 3.1 Characterization of DSCP

**FIGURE 1:**
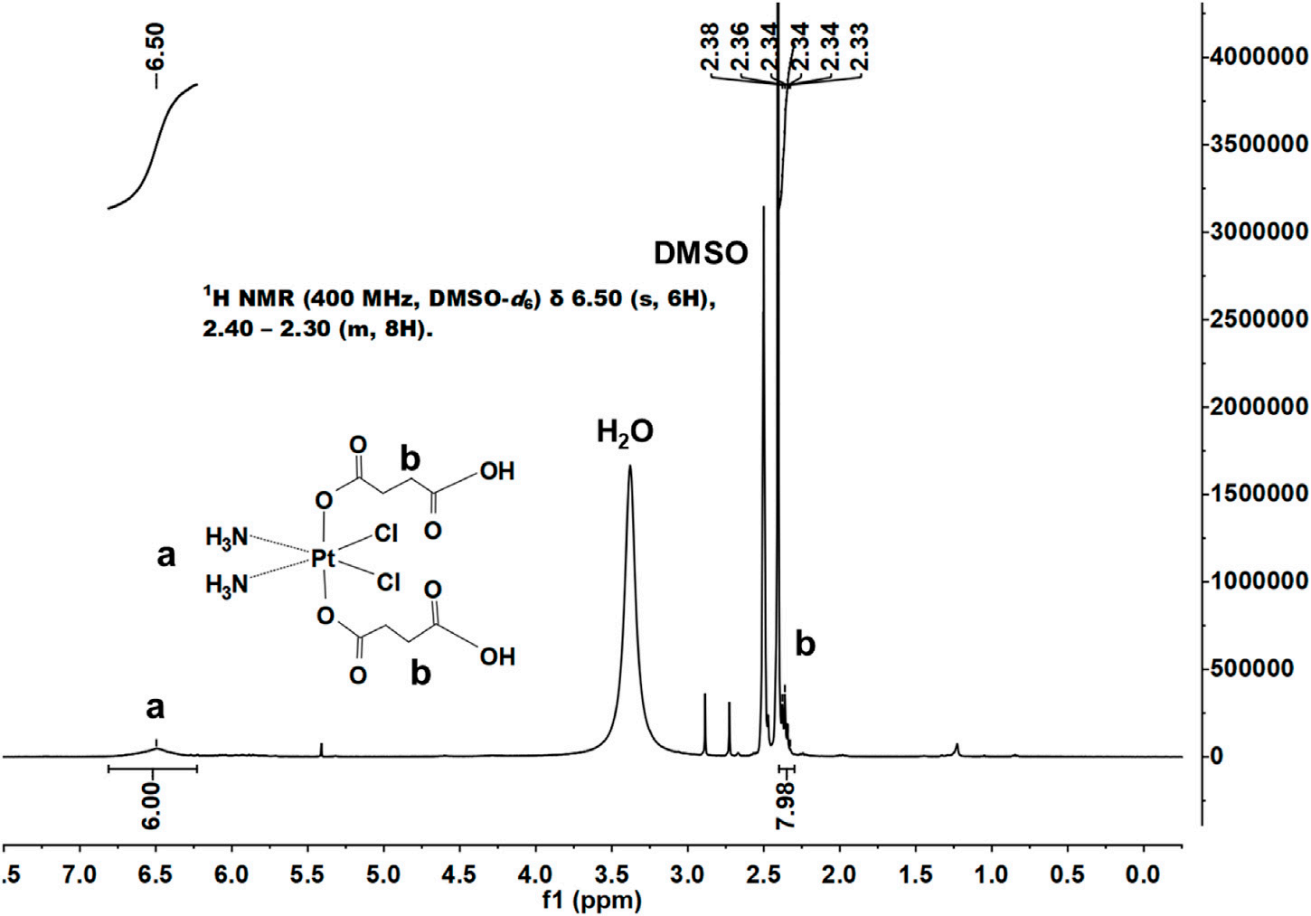
^1^H NMR spectrum of c,c,t-[Pt (NH_3_)_2_Cl_2_(O_2_CCH_2_CH_2_CO_2_H)_2_] in DMSO-d_6_.

**FIGURE 2:**
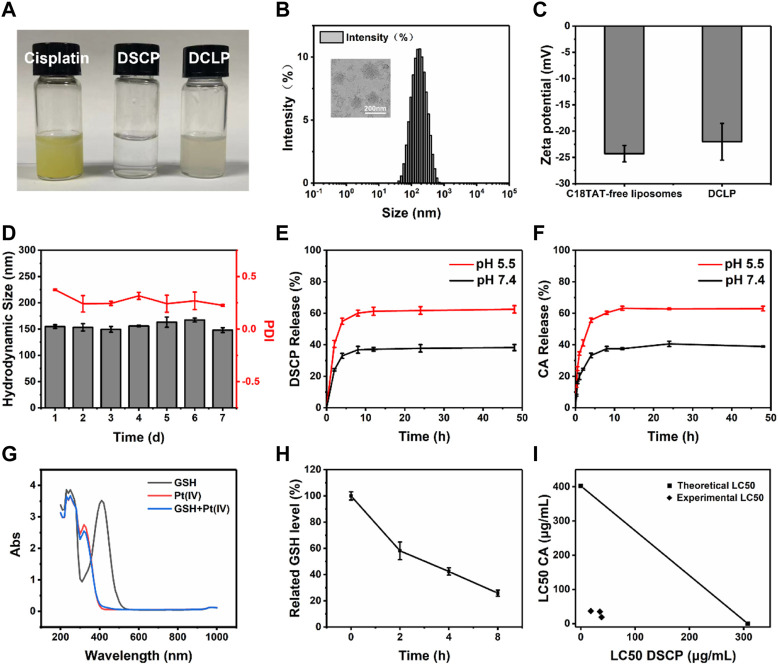
Characterization and responsiveness of DSCP and DCLP. **(A)** Photos of cisplatin, DSCP and DCLP. **(B)** TEM image and size distribution of DCLP. **(C)** Zeta potential of DCLP. **(D)** Stability of DCLP in PBS. Scale bar = 200 nm. **(E)** Release of DSCP under different pH conditions. **(F)** Release of CA under different pH conditions. **(G)** GSH reactivity of DSCP detected by the glutathione assay kit. **(H)** For the analysis of GSH depletion in cells by DSCP, the initial GSH content was set as a control group (*n* = 3). **(I)** Isobologram showing that DSCP and CA have additive effects in tumor cells. Using the LC50 of both drugs for the graph, straight contours similar to the theoretical additive lines indicate additive effects.

We successfully synthesized DSCP according to a previous method, and the results of ^1^H NMR supported the assigned structure (Figure 1). According to the Ellman method, 5,5ʹ-dithio-bis-(2-nitrobenzoic acid) (DTNB) can react with the side chain of free sulfhydryl groups on a protein to form an S-S bond between the protein and 2-nitro-5-mercaptobenzoic acid (TNB) residues, thereby producing TNB and GSSG (L-glutathione oxidized). TNB shows a characteristic absorption peak at 412 nm; thus, the GSH content can be quantified by the change in the absorbance value at 412 nm. With the indication of TNB, the association between DSCP and GSH reduction was determined by measuring the height of the absorption peak. After co-incubation with the prepared DSCP, UV-Vis detection revealed that the absorption peak of TNB at 412 nm disappeared, thus confirming that the presence of DSCP effectively reduced GSH level (Figure 2G). This situation indicated that the redox level in the cells was disrupted. Triplicate samples of 10^6^ cells were then co-incubated with DSCP and then assayed for GSH at 0, 2, 4, 6 and 8 h using the Glutathione Assay Kit with BSA as reference. The assay was performed according to the method recommended by the manufacturer. The standard and control sample cuvettes were placed in a spectrophotometer and the decrease in absorbance at 412 nm was monitored over time. Concentrations were converted to nmol/mg protein and the relative amount and standard deviation of GSH per hour was calculated separately. The results are shown in Figure 2H. ^1^H NMR: (400 MHz, DMSO-d_6_) δ 6.50 (s, 6H), 2.40–2.30 (m, 8H).

### 3.2 Characterization of DCLP liposomes

Having identified that DSCP and CA have a synergistic effect on each other (Figure 2I), DCLP liposomes were successfully prepared by the thin film hydration method (Figure 2A). The liposomes were diluted with deionized (DI) water to achieve a concentration of 2 mg/mL. The mixture was then added dropwise onto a carbon film-covered copper mesh (400 mesh) for 15 min at room temperature and dried. Subsequently, the external shape of the liposomes was evaluated via a TEM. The morphology of DCLP was relatively regular and spherical with uniform dispersion, and the average particle size of DCLP was approximately 120 nm (Figure 2B). We then measured the particle size distribution and zeta potential of liposomal nanoparticles by using a Malvern Zetasizer (Nano ZS), the hydrodynamic size and zeta potential of DCLP were 155 ± 3.6 nm and −21.5 ± 0.5 mV, respectively (Figures 2B, C). We also measured the stability of the nanoparticles with a particle size meter. The nanoparticles were maintained in PBS at pH 7.4 for 7 days, and their particle size and polymer dispersity index (PDI) were examined daily. The final results showed that the nanoparticles were stable in size, and their PDI was always below 0.4 (Figure 2D); thus, it was concluded that the developed liposomes could stay stably in PBS up to a certain period of time.

### 3.3 Encapsulation efficiency and drug loading

3.3.1 CA: The CA standard curve was first established. A CA solution (12.5 μg/mL) was prepared using chloroform as the solvent, and the absorbance of the solution was subsequently measured using a UV-Vis spectrophotometer. Absorption peaks were detected at 286 nm, which were selected as the spectral peak of the standard curve. Next, standard solutions with mass concentrations of 12.5, 6.25, 3.125, and 1.5625 μg/mL were prepared. The absorbance values were measured separately for each concentration of CA solution at 286 nm, and the absorbance value y was linearly fitted to the mass concentration X. The regression equation was as follows: y = 0.003x - 0.0058, *R* ^2^ = 0.9959; this indicated a good linear relationship between CA and the absorbance value in the measured concentration range. The liposomes were removed from the precipitate by centrifugation at 14,000 rpm for 10 min. The supernatant was extracted by adding chloroform, and the absorbance value of the supernatant at 286 nm was measured for free CA that failed in being packed into the liposomes. The CA extracted in the supernatant with chloroform after centrifugation of the liposomes is the fraction that failed to integrate successfully with the liposomes. We derived the amount of CA corresponding to the absorbance value and compared it with the total amount of CA added, and the difference obtained was the amount of CA encapsulated in the liposome.

3.3.2 DSCP: For quantitative measurement, lyophilized DCLP was digested by adding 500 μL of aqua regia for 12 h. On the following day, 9.5 mL of 2% nitric acid was added, and the platinum content was determined by inductively coupled plasma-mass spectrometry (ICP-MS). With the amount of drug added determined, the drug loading ratios (DL) and encapsulation efficiency (EE) of the two drugs were calculated by measuring the drug content in the DCLP.

Finally, the EE (%) and DL (%) of X (X refers to a certain drug) were calculated using the following equations:

$$DL\%=\frac{amount\;of\;X\;loaded\;in\;Liposome\;suspension}{Total\;amount\;of\;X\;and\;excipents\;in\;liposome\;suspension}\times 100$$

$$EE\%=\frac{amount\;of\;X\;loaded\;in\;Liposome\;suspension}{Amount\;of\;X\;added\;during\;compositing}\times 100$$

Based on the formula, we calculated the amount of DSCP and CA loaded in the liposomes as shown in the table below.

### 3.4 Determination of drug release capacity *in vitro*

The efficiency of the developed liposomes to release CA and DSCP was determined by UV-Vis spectrophotometry and ICP-MS, respectively. Two equal masses and volumes of DCLP were lyophilized and added to PBS of different pH values (5.5 and 7.4) (Feng et al., 2017; Chen et al., 2018); the solutions were then placed in pre-prepared dialysis bags, and the bags were sealed at both ends with a cling film and secured with clips. The dialysis bags were then placed into two centrifuge tubes containing PBS having the same pH as that of PBS in the dialysis bag. The centrifuge tubes were placed in a constant temperature shaker at 120 rpm and shaken at 37 °C for 48 h. The release behavior of CA in PBS with different pH values was investigated by the dialysis method. The outer layer of liquid was aspirated separately, chloroform was added to extract CA from the solution, and the absorbance was measured at 286 nm to determine the amount of CA released. The concentration of Pt was measured by ICP-MS (*n* = 3). During dialysis, we collected the liquid present outside the dialysis bag and added chloroform for extraction. The absorbance values were measured using a UV-Vis spectrophotometer. The results showed that the CA release in PBS at pH values of 5.5 and 7.4 was 63.02% and 38.96% (Figure 2F), respectively. The residual liquid present outside the dialysis bag was analyzed by ICP-MS, and the corresponding DSCP release values were 59.26% and 36.26% at pH 5.5 and 7.4 (Figure 2E), respectively.

### 3.5 Assessment of cytotoxicity of DCLP

**FIGURE 3:**
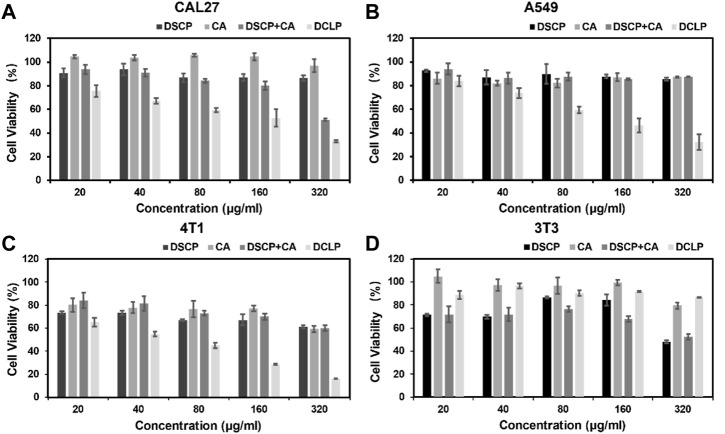
*In vitro* cell viability of **(A)** CAL27, **(B)** A549, **(C)** 4T1, and **(D)** 3T3 cells after 24 h of co-culture with the indicated reagents was detected by MTT assay (*n* = 4).

Four different cell lines in the logarithmic growth phase were used to validate the *in vitro* cytotoxicity and bioavailability of DCLP. The cells were cultured in DMEM or 1640 containing 10% FBS at 37 °C in a humidified atmosphere with 5% CO_2_. Subsequently, the cells were seeded onto 96-well plates at the density of 1 × 10^4^ cells/mL and incubated for 24 h. After the incubation period, DCLP nanoparticles were added to a fresh medium at successive concentrations (0, 20, 40, 80, 160 and 320 μg/mL, mass concentration of DCLP.) to replace the original medium and incubated for another 24 h. In addition, the drug concentrations of DSCP and CA are known from pre-determined drug loading rates. Cell viability was determined by the standard methylthiazolyl tetrazolium assay (MTT). The toxicity of DCLP was verified using cytotoxicity assays (Figure 3). The survival rate of cells incubated with liposomes loaded with both drugs was the lowest, thus indicating superior tumor inhibition activity of DCLP *in vitro*. Compared with the results of the sole drug application of either DSCP or CA, the antitumor effect of the combination of the two drugs loaded on liposomes was significantly improved, thus indicating that DSCP and CA has a clear anti-tumor capacity *in vitro*. The modification of the C18-TAT peptide on the surface of the liposomes made them more capable of releasing drugs in the acidic TME, and given the alkaline pH of normal tissue, DSCP had less adverse effects on normal tissue cells with CA, which was also verified in the 3T3 cell line. In addition, we compared the cytotoxicity of C18TAT-FREE liposomes and DCLP in 4T1 cells. The results (shown in Figure 5A) are consistent with the previous analysis and confirm that the selective killing of tumor cells by DCLP correlates with its surface modification of C18TAT.

### 3.6 Apoptosis and intracellular uptake measurement by flow cytometry

**FIGURE 4:**
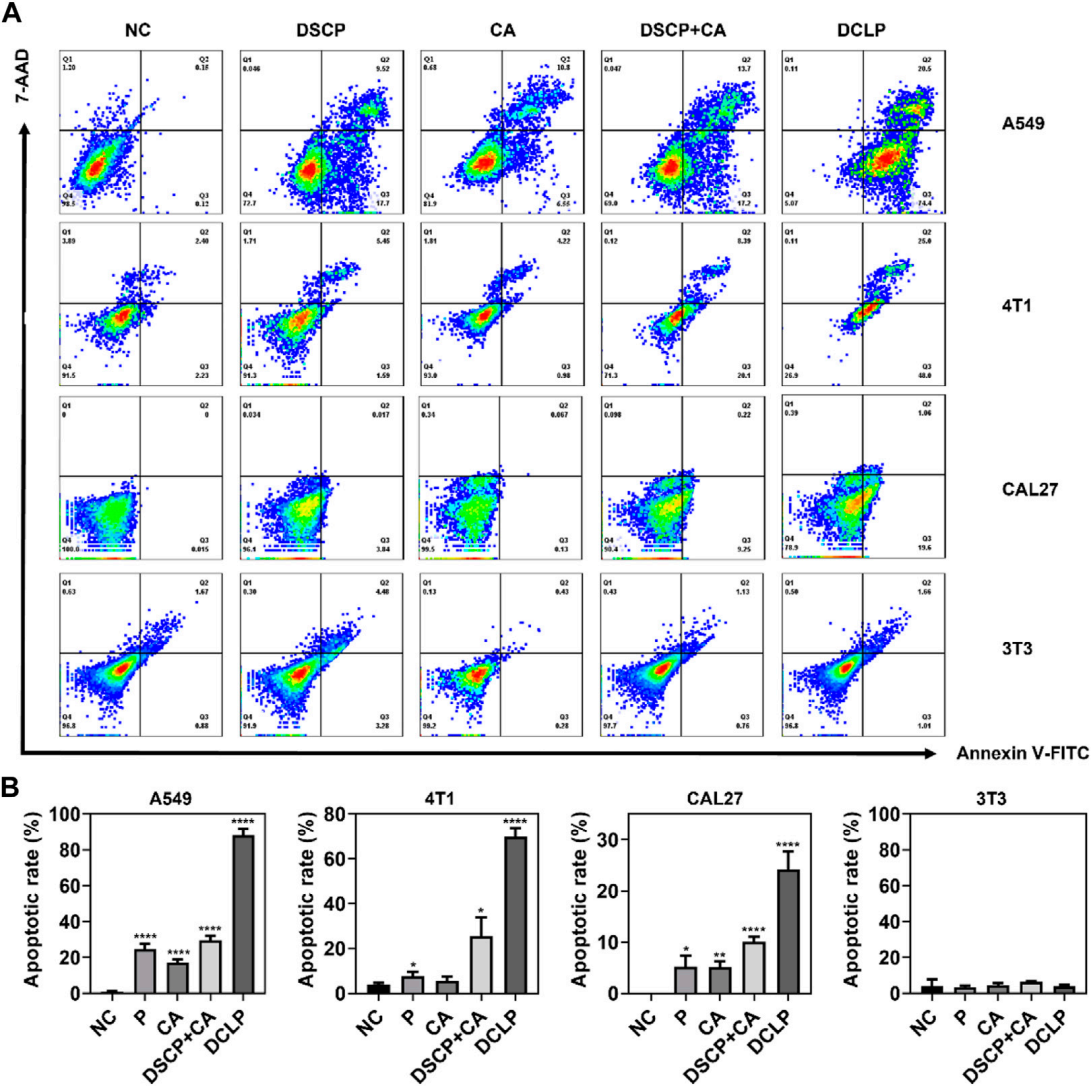
**(A)** Apoptosis rates of A549, CAL27 and 4T1 after co-incubation with DCLP for 12 h as measured by flow cytometry. **(B)** The quantified results of apoptosis rates. Data were given as mean ± S.D. (*n* = 3). One-way ANOVA analysis with Tukey’s multiple comparison test was used. Significance is presented as ****p < 0.0001.

**FIGURE 5:**
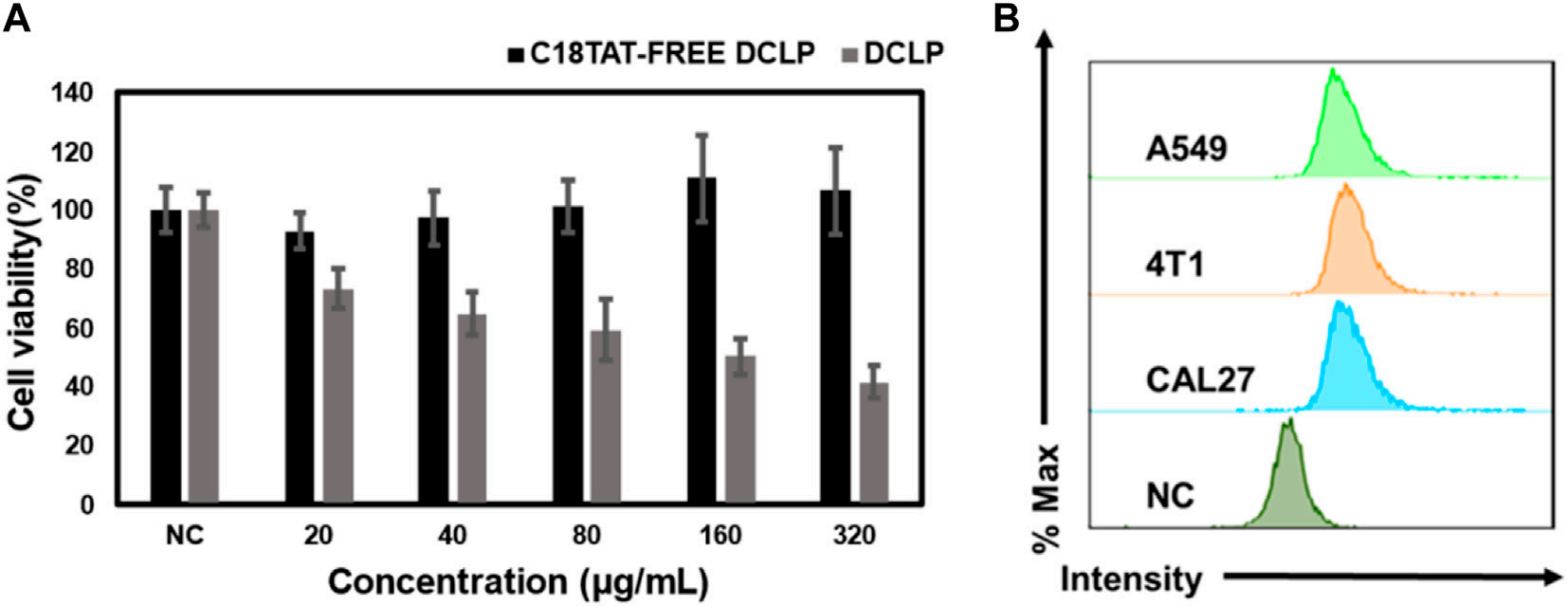
**(A)** Comparison of the killing effect of C18TAT-FREE liposomes and DCLP on 4T1 cells. **(B)** Uptake rates of A549, CAL27 and 4T1 after co-incubation with DCLP for 12 h as measured by flow cytometry. Data were given as mean ± S.D. (*n* = 3). One-way ANOVA analysis with Tukey’s multiple comparison test was used. Significance is presented as ****p < 0.0001.

The induction of apoptosis after co-incubation with DCLP was assessed in different cell lines, all the cells were seeded onto 6-well plates at the density of 5 × 10^5^ cells per dish and cultured until confluence. DCLP was then co-incubated with the cells for 12 h. Subsequently, the cells were collected and treated with Annexin V-FITC and 7-AAD for 15 min at room temperature in dark. The cell apoptosis rate after different treatments was measured by flow cytometry. The results were shown In Figures 4A, B, where the same trend of cell death is the same as that noted in the cytotoxicity assay. After a 24 h drug treatment, The mortality rates of all three tumor-associated cell lines involved were increased as compared with that of the control group (We use the 3T3 cell line in this experiment to refer to normal cells that are distinct from tumor cells), thereby demonstrating the general toxicity of DCLP to different tumors. Intracellular uptake of A549, 4T1 and CAL27 was also measured by flow cytometry and we loaded FITC into liposomes using a method of preparing DCLP to determine the uptake of DCLP in tumor cells. Figure 5B shows that all three cells showed successful uptake of DCLP.

### 3.7 Calcein-AM and propidium iodide staining

**FIGURE 6:**
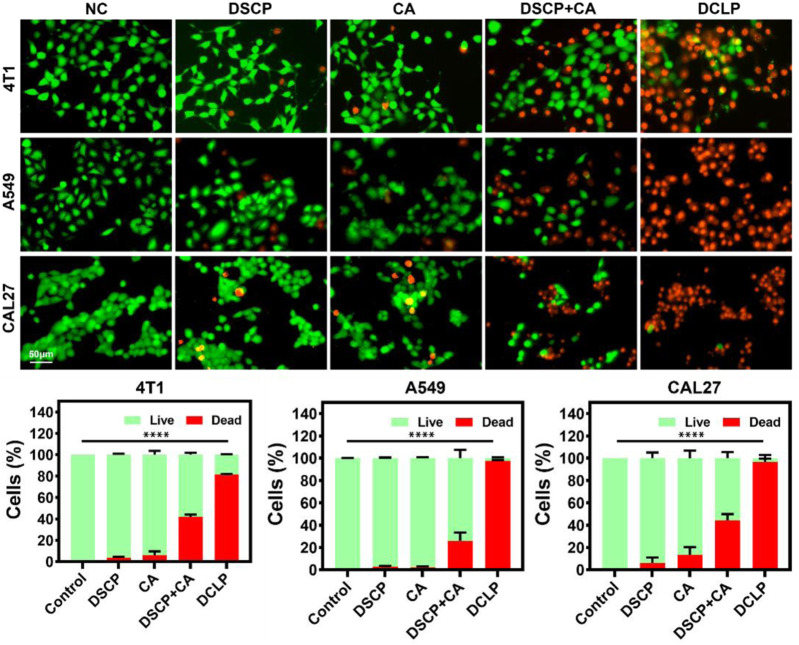
*In vitro* live (green) and dead (red) assay. Data were given as mean ± S.D. (*n* = 3). One-way ANOVA analysis with Tukey’s multiple comparison test was used. Significance is presented as ****p < 0.0001.

The cytotoxicity of the optimized liposomes was observed by live-dead staining assay. After treatment with DCLP (25 μg/mL) for 24 h, the cells were washed with PBS. After washing, the cells were placed in a fresh medium, 1 μM calcein-AM and 1 μM propidium iodide (PI) were added, and the cells were incubated for 30 min. The fluorescent cells were observed with the EVOS M7000 3D digital confocal cell imaging analysis system, and the images of cell fluorescence were captured. As shown in Figure 6, in the dark field view, the DCLP group had the strongest red fluorescence, indicating that the combination of DSCP and CA led to significant cell death. It was within our anticipation that, when compared with the NC group, the DSCP and CA groups showed different degrees of enhanced red fluorescence; Furthermore, it’s worth noticing that the best therapeutic effect turned out when the two drugs were applied simultaneously.

### 3.8 Measurement of ROS production

**FIGURE 7:**
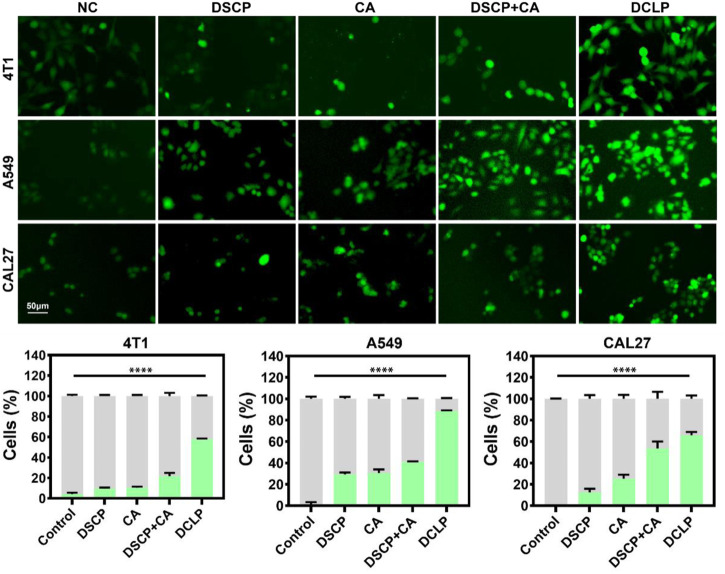
*In vitro* DCFH-DA assay. Data were given as mean ± S.D. (*n* = 3). One-way ANOVA analysis with Tukey’s multiple comparison test was used. Significance is presented as ****p < 0.0001.

ROS production was detected by dichlorofluorescein-diacetate assay (DCFH-DA). The cells were treated with DCLP (25 μg/mL) for 24 h and then washed with PBS. After the cells were washed three times, they were incubated with 10 μM DCFH-DA for 20 min at 37°C in dark. Subsequently, the stained cells were observed with the EVOS M7000 3D digital confocal cell imaging analysis system, and cell fluorescence images were captured. As shown in Figure 7, the DCLP group showed the most apparent green fluorescence in the dark field view, thus indicating that the DSCP and CA combination induced a significant increase in ROS accumulation level, which in turn enhanced the efficacy of ROS in killing tumor cells. Compared with the NC group, both the DSCP and CA groups showed different degrees of enhanced green fluorescence; this finding indicated that though the application of a single drug could also elevate intracellular ROS level, the effects were not as ideal as those by the combination of the two drugs.

## 4 Discussion

In the present study, DCLP was successfully prepared using the pH-responsive peptide C18-TAT-modified liposomes loaded with the lipophilic drug CA and the hydrophilic drug DSCP as carriers. This approach not only improved the stability and biocompatibility of the drug but also optimized drug metabolism and reduced drug toxicity. Based on previous studies, it is reasonable to assume that the optimized structure of liposome is more stable and shows more uniform and regular morphology in transmission electron microscopy, and it bears an extended resting time after the synthesis. The particle size measurement results indicated that the optimized dual-drug liposomes were in small size and had narrow size distribution; these properties facilitated the entry of DCLP into cancer cells and the subsequent responsive release of CA and DSCP, thereby increasing the intracellular accumulation of drugs and enhancing their antitumor effects. Compared with the results from transmission electron microscopy, the average hydrated particle size measured via a particle size meter was larger than the average size of the liposomes that had been dried when they were observed under a TEM; this might be due to the loss of the hydrated layer on the liposome surface. The results of an *in vitro* drug release assay showed that up to 61.31% CA was released on the average after 12 h in a pH 5.5 environment; and the release rate of DSCP was 63.21% on the average after 12 h These outcomes were higher than those reached in a pH 7.4 environment. These finding indicated that DCLP has an acid-responsive drug release property, and it can function in an acidic TME, with a reduction of drug release in normal tissues. *In vitro* experiments confirmed that DCLP has low toxicity to normal tissues while maintaining its potent tumor cell-killing effects.

Compared to conventional cisplatin chemotherapy treatment, the combination of CA and DSCP not only reduces the toxicity of cisplatin to normal cells but also overcomes tumor resistance to a certain extent, thereby enhancing the antitumor effect. Most importantly, as an amplifier of oxidative stress, the nanosystem significantly enhanced CA-induced ROS production, thus promoting ROS accumulation through GSH depletion and severely disrupting redox homeostasis in tumor cells. This strategy of combining ROS therapy with GSH depletion can effectively promote the use of DCLP nanodrugs to modulate ROS in combination with chemotherapy in synergistic antitumor applications. In addition to the approach of ROS accumulation combined with chemotherapy treatment used in the present study, other ROS-related antitumor approaches could also be employed in this therapeutic strategy, including photodynamic therapy, acoustic therapy, and chemodynamic therapy, which are used to trigger cell death by targeting the delivery of specific active substances to cancer tissues and generating large amounts of ROS under the activation of light, ultrasound, or chemical reactions. The efficacy of these approaches is, however, limited by the scavenging of ROS by high GSH levels in tumor cells, and therefore by combining GSH depletion with various ROS therapies, both could serve as promising and effective strategies to improve antitumor efficacy.

## 5 Conclusion

In the present study, we successfully synthesized a liposomal drug delivery platform that can co-deliver DSCP and CA and confirmed its acid-responsive release property through morphological structure evaluation and drug release characterization studies. *In vitro* cytotoxicity experiments demonstrated the synergistic effect of DSCP and CA in killing tumor cells. The possible mechanisms are hypothesized as follows: (1) the activation of DSCP requires GSH depletion upon the uptake of DCLP into cells, as compared to the ROS-producing effect of CA itself. This increases the antitumor effect of ROS to some extent; (2) activation of DCLP requires GSH depletion, which increases GSH depletion in the cell, and (3) a decline in GSH content prevents cisplatin resistance to a certain extent and enhances the cell-killing effect of cisplatin. The present study provides a practical strategy for enhancing ROS-mediated oxidative therapy by combining two drugs for synergistic antitumor treatment.

**TABLE 1:** Encapsulation efficiency and drug loading of DCLP (Mean ± SD,*n* = 3).

Liposome	DSCP		CA	
	EE%	DL%	EE%	DL%
DCLP	18.57 ± 0.19	0.67 ± 0.09	36.38 ± 0.13	1.29 ± 0.04

## Data Availability

The original contributions presented in the study are included in the article/supplementary material, further inquiries can be directed to the corresponding authors.
